# The Effect of Interleukin-10 Immunotherapy on Renal Ischemia-Reperfusion Injury: A Systematic Review and Meta-Analysis of Preclinical Studies

**DOI:** 10.3390/ijms25116231

**Published:** 2024-06-05

**Authors:** Apostolos Prionas, Karim Hamaoui, Konstantinos Vanezis, Vikash Reebye, Nagy Habib, Vassilios Papalois

**Affiliations:** Department of Surgery and Cancer, Imperial College London, London W12 0HS, UK; apostolos.prionas@nhs.net (A.P.); karim.hamaoui@nhs.net (K.H.); konstantinos@minatx.com (K.V.); v.reebye@imperial.ac.uk (V.R.); nagy.habib@imperial.ac.uk (N.H.)

**Keywords:** ischemia-reperfusion injury, interleukin-10, kidney transplantation

## Abstract

Renal ischemia-reperfusion is a common cause of acute kidney injury leading to significant morbidity and mortality. There are no effective treatments available in clinical practice. This meta-analysis aims to assess the effect of IL-10 immunotherapy on renal ischemia-reperfusion injury. Medline, Embase, Cochrane-library, Google Scholar and clinicaltrials.gov were searched up to 31 March 2023. Preclinical and clinical interventional studies investigating IL-10 immunotherapy for renal ischemia-reperfusion were eligible for inclusion. The primary endpoint was renal function (serum creatinine) following ischemia-reperfusion. The secondary endpoints included mitochondrial integrity, cellular proliferation, regulated cell death (TUNEL assay), expression of inflammatory cytokines (TNF-α, IL-6 and IL-1β), M1/M2 macrophage polarization, tissue integrity (tubular injury score), long-term kidney fibrosis (fibrotic area %) and adverse events (pulmonary toxicity, cardiotoxicity hepatotoxicity). The search returned 861 records. From these, 16 full texts were screened and subsequently, seven animal studies, corresponding to a population of 268 mice/rats, were included. Compared to the control treatment, IL-10 immunotherapy reduced serum creatinine more effectively within 24 h of administration (95% CI: −9.177, −5.601, I^2^ = 22.42%). IL-10 immunotherapy promoted mitochondrial integrity and cellular proliferation and reduced regulated cell death (95% CI: −11.000, −4.184, I^2^ = 74.94%). It decreased the expression of TNF-α, IL-6 and IL-1β, led to M2 polarization of the local macrophages, reduced tubular injury score (95% CI: −8.917, −5.755, I^2^ = 22.71%), and long-term kidney fibrosis (95% CI: −6.963, −3.438, I^2^ = 0%). No adverse outcomes were captured. In Conclusion, IL-10 immunotherapy safely improves outcomes in animal models of renal ischemia-reperfusion; the translational potential of IL-10 immunotherapy needs to be further investigated in clinical trials.

## 1. Introduction

Renal ischemia-reperfusion injury (IRI) is the most common cause of acute kidney injury (AKI) perioperatively in patients receiving solid organ transplants. It is associated with high morbidity and mortality [1]. In the kidney transplant recipient, renal IRI may lead to primary non-function or delayed graft function and can increase the allograft’s immunogenicity favoring T-cell and antibody-mediated rejection [2]. It can also result in chronic allograft dysfunction secondary to interstitial fibrosis and tubular atrophy (IFTA) [2]. 

Most of the damage resulting from renal IRI occurs upon reperfusion and upon the subsequent stage of sterile inflammation. This damage is facilitated by the innate and the adaptive immune system. Various cytokines play a crucial role in shaping the local immune microenvironment, balancing between damage (inflammation and fibrosis) and repair (regeneration). Certain cytokines such as interferon γ (IFN-γ), tumor necrosis factor α (TNF-α), and interleukin 1β (IL-1β) tend to promote inflammation, while others like interleukin-10 (IL-10) tend to suppress the immune response and support regeneration [2].

The IL-10 is a T helper type 2 (Th2) cytokine produced by cells of innate and adaptive immunity: natural killer cells, macrophages, dendritic cells, Th2 and T-regulatory cells, B cells, including B-regulatory cells [3]. In the kidneys, it is also produced by the mesangial and the endothelial cells. IL-10 was found to inhibit the T helper type 1 (Th1) response, the activation and differentiation of antigen-presenting cells, such as dendritic cells and macrophages by down-regulation of the expression of major histocompatibility complex class II and co-stimulatory molecules and decrease in the secretion of pro-inflammatory cytokines [3]. It is also thought to promote a shift to an M2 phenotype of the macrophages leading to advanced angiogenesis, anti-inflammatory action, wound healing and tissue remodeling [4]. At a cellular level, IL-10 signaling is followed by the activation of the Janus kinases and the members of the signal transducer and activator of the transcription family. This results in enhanced transcription of genes associated with cell cycle progression (ki67 and others) and in the down-regulation of factors associated with regulated cell death (i.e., caspase-3 and others) [3]. In our experience, the adaptive immune response, the local inflammation and the regulated cell death are key determinants of outcomes in renal IRI [5]. 

Moreover, IL-10 has an autocrine effect in the renal mesangial cells acting as a growth or activation factor. The mesangial cells are principal regulators of kidney function through the regulation of the extracellular matrix, the size of the capillary loops and the glomerular filtration rate [3]. For the normal kidney function to be maintained, the mesangial activity, cell cycle and proliferation should be within strict limits. Mesangial cells’ activation or proliferation can, in principle, result in renal structural intraglomerular and tubulointerstitial changes. Glomerulosclerosis and tubulointerstitial fibrosis are pathological changes that can lead to chronic/end-stage renal disease [3].

On the one hand, it appears that through its immunomodulatory, anti-inflammatory and anti-apoptotic functions, IL-10 immunotherapy could have a protective role against renal IRI. On the other hand, there is the risk of activation or proliferation of the renal mesangial cells and the induction of fibrosis that might have a lasting detrimental impact. Thus, it is currently unclear whether IL-10 immunotherapy could be beneficial in the context of renal IRI. Our preliminary hypothesis is that in the acute setting of kidney IRI, IL-10 immunotherapy exerts a renoprotective and anti-fibrotic rather than a nephrotoxic and profibrotic effect. Therefore, the objective of the present study is to systematically review the effect of IL-10 immunotherapy on renal ischemia-reperfusion injury and make recommendations with a view to inform future translational work for the development of targeted treatments.

## 2. Materials and Methods

### 2.1. PICO Research Question

To address this objective, a targeted research question was developed. The PICO (population, interventions, comparison, outcomes) breakdown of this research question is outlined below:

- Population:
  I. Animals or humans undergoing renal ischemia-reperfusion (animal or human subjects).
  II. Animal or human kidneys undergoing ischemia-reperfusion (animal or human tissues).
- Interventions:
  I. Exogenous administration of IL-10.
- Comparison:
  I. No exogenous administration of IL-10.
- Outcomes:
  I. Primary: renal function following renal IRI.
  II. Secondary: mitochondrial integrity of tubular epithelial cells (TECs); TECs’ proliferation; regulated TECs’ death; tissue expression of inflammatory cytokines; M1/M2 macrophage polarization; tissue integrity and inflammation; long-term kidney fibrosis; and adverse treatment outcomes.

### 2.2. Eligibility Criteria

The inclusion and exclusion criteria for our systematic review are presented in Table 1.

The present systematic review followed the best practice guidelines for the preparation, conduct and publication of systematic reviews of animal and human interventional studies [6,7]. A full list of the complete forms of the abbreviations used throughout the manuscript, including the tables, can be found in the Abbreviations section.

### 2.3. Search Strategy, Study Selection and Data Collection Process

In order to perform this systematic review, after establishing a detailed and thorough search strategy, Embase, Medline, Cochrane Library, Google Scholar databases and clinical trials.gov registry were screened up to 31 March 2023. The reference lists of the identified studies were also screened. Databases screening and studies’ selection process followed the PRISMA (preferred reporting items for systematic reviews and meta-analyses) and the CAMARADES (collaborative approach to meta-analysis and review of animal data from experimental studies) guidelines and were performed by two reviewers working independently [6,8]. There were no language or publication date restrictions. All disagreements were resolved by the vote of a third independent reviewer. The full search strategy for one of the databases screened (Embase) is presented in Table S1 (Supplementary Material). Data were extracted using a pre-defined data collection spreadsheet. When needed, we contacted the corresponding authors of the included studies for clarification.

### 2.4. Data Items

Data were extracted on:

A. Study and Population Characteristics:
  (1) Study ID (authors, year)
  (2) Study design (qualitative data)
  (3) Study subjects (qualitative data)
  (4) Study size (number of subjects)
  (5) Renal IRI induction protocol (qualitative data)
  (6) Warm Ischemia Time (WIT) (in minutes)
  (7) Study groups (qualitative data)
B. Interventions’ Characteristics:
  (1) Form of IL-10 administered (qualitative data)
  (2) Carriers/Delivery Vehicles (qualitative data)
  (3) Timing of first IL-10 administration in reference to kidney reperfusion (in hours)
  (4) Route of IL-10 administration (qualitative data)
  (5) Dose of IL-10 administered (in μg)
  (6) Frequency of IL-10 administration (qualitative data)
  (7) Control intervention (qualitative data)
C. Outcomes:

After identifying the most appropriate experimental and control groups for the present systematic review (consensus between reviewers), data were extracted on:

I. Primary Outcome:
  (1) Serum Creatinine (mg/dL) within 24 h of treatment administration.
II. Secondary Outcomes:
  (1) TECs’ mitochondrial integrity within 24 h of treatment administration (qualitative data)
  (2) TECs’ proliferation, including expression of ki-67 and proliferating cell nuclear antigen (PCNA), within 24 h of treatment administration (qualitative data)
  (3) Number of terminal deoxynucleotidyl transferase dUtp nick-end labelling (TUNEL) positive cells/high power field (HPF) within 24 h of treatment administration
  (4) Expression/activity of caspase-3 within 24 h of treatment administration (qualitative data)
  (5) Expression of TNF-α, interleukin-6 (IL-6) and IL-1β within 24 h of treatment administration (qualitative data)
  (6) M1/M2 polarization of macrophages including expression of inducible Nitric oxide synthase (iNOS) and cluster of differentiation 206 (CD206) (qualitative data)
  (7) Tubular Injury Score within 24 h of treatment administration
  (8) Long-term kidney fibrosis (fibrotic area %) following renal IRI
  (9) Long-term cardiotoxicity following treatment administration (qualitative data)
  (10) Long-term pulmonary toxicity following treatment administration (qualitative data)
  (11) Long-term hepatotoxicity following treatment administration (qualitative data).

### 2.5. Identification of Risk of Bias in Individual Studies

The risk of bias in the individual studies was assessed in an outcome and study level via:

- Narrative critical appraisal of each study and bias identification.
- The use of systematic review centre for laboratory animal experimentation (SYRCLE) risk of bias (RoB) tool for animal studies [9].

To assess risk of selective reporting bias we compared outcomes and analyses pre-specified in study registers or protocols with the results available in the study reports.

### 2.6. Summary Measures and Synthesis of the Results

For the quantitative synthesis of the results, the standardized mean difference was used. The meta-analyses results were reported using 95% confidence interval (CI) and displayed graphically using forest plots. The results of the studies were combined in meta-analyses using standard random effects models, from which estimates of the average standardized mean difference were obtained, with 95% CI. To assess heterogeneity, I^2^ was obtained. Where possible we further investigated the heterogeneity encountered with subgroup meta-analyses. Multiple sensitivity analyses were performed to confirm the robustness of the synthesized results. We explored whether the intervention or the control group selection (i.e., the different doses/delivery vehicles of IL-10, or the control interventions with normal saline resuscitation/vehicle treatment), the primary outcome measures (i.e., serum creatinine vs. Blood Urea Nitrogen—BUN), and timing of assessments influenced our results. None of these analyses contradicted our findings, indicating the reliability of our results. Detailed results of the sensitivity analyses are not reported as they align with our findings. All analyses were conducted in the openmetaanalyst software version 10.12 with a significance level of 0.05.

### 2.7. Identification of Risk of Bias across Studies

The risk of bias across studies was assessed (narrative assessment). The risk of bias across studies analysis was further complemented with the application of the grading of recommendations assessment, development and evaluation (GRADE) methodology. The GRADE methodology was tailored for systematic reviews of animal studies [10]. The quality of the generated evidence was graded based on five factors: risk of bias, indirectness, inconsistency, imprecision, and publication bias. Furthermore, in order to mitigate the risk of publication bias in our study, our search strategy included grey literature screen, with no language and time restrictions.

## 3. Results

### 3.1. Study Selection

**Figure 1:**
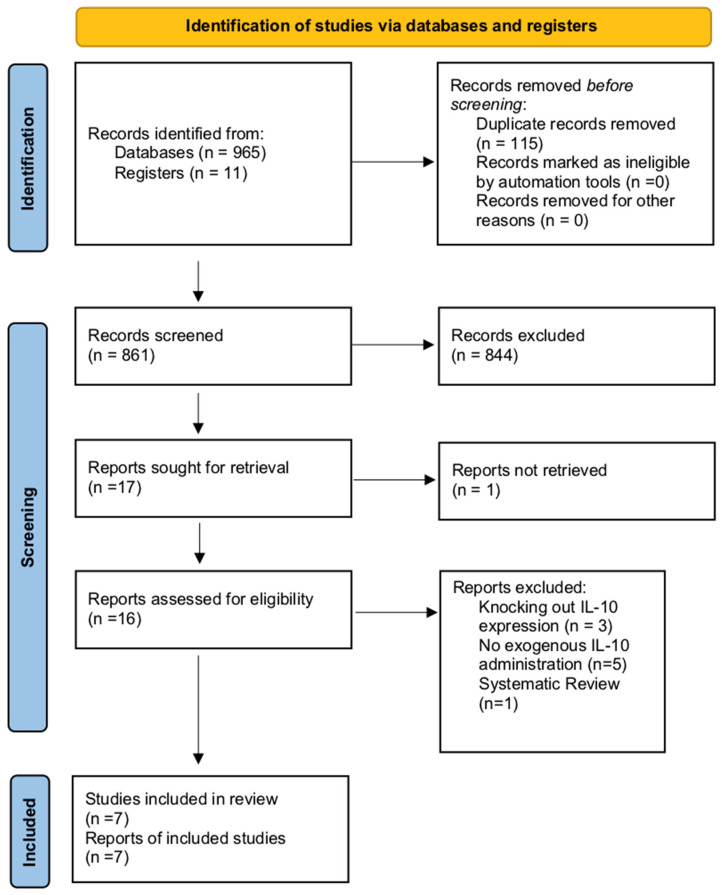
Systematic review flow diagram.

The study’s flow diagram is presented in Figure 1. In total, 965 records were identified through database screening and 11 were identified from registers. After the elimination of duplicate entries, 861 records were screened. A total of 844 records were excluded. Sixteen full-text articles were assessed for inclusion in the present review. Nine studies were excluded and the reasons for exclusion are presented in the diagram [4,11,12,13,14,15,16,17,18]. Overall, seven preclinical animal studies were included in the systematic review and the meta-analysis [19,20,21,22,23,24,25]. No clinical studies investigating IL-10 immunotherapy for renal IRI were identified.

### 3.2. Risk of Bias and Study Characteristics

The included studies’ SYRCLEs risk of bias summary assessment is presented in Table 2. No standardized processes for the random allocation of animals in groups and for the blinding of the researchers to the allocations were mentioned in the included studies’ reports. The risk of selection bias was deemed overall unclear in the studies by Wang X et al., Soranno D. et al., Jung M, et al. and Deng J. et al. due to a lack of reporting the animals’ baseline characteristics (gender, age and weight) [21,22,23,25]. None of the included studies scored high in any of the SYRCLE’s RoB domains.

The included studies’ characteristics are presented in Table 3. All the studies were in vivo animal studies in different strains of mice (*C57BL/6* and *BALB/c*) and rats (*Sprague-Dawley*) of similar age and weight. The disease induction methodology was fairly common across the studies (midline laparotomy followed by either bilateral or unilateral renal pedicle clamping). There was, though, significant variability in warm ischemia time (22–45 min). The different intervention and control groups for each study can be found in Table 3, with the most appropriate (target) experimental and control groups for the present systematic review highlighted in bold.

A summary of the target IL-10 immunotherapy and control interventions across the included studies is presented in Table 4. As illustrated in the table, there was significant heterogeneity between both the IL-10 immunotherapy interventions (different forms of IL-10 administered, different delivery vehicles, different delivery strategies and doses) and the control interventions (normal saline treatment vs. vehicle treatment).

### 3.3. Results of Individual Studies

The results of the included studies are presented in Table 5A–C. The effects of IL-10 immunotherapy on renal function and the adverse outcomes are presented in Table 5A. In the majority of the included studies, serum creatinine was measured using chemical methods (Jaffe method) [22,23,24,25]. Gong H. et al. measured serum creatinine using isotope dilution liquid chromatography–mass spectrometry (IDMS) [19]. Two of the included studies did not report the method of serum creatinine measurement [20,21]. The effects on the TEC following renal ischemia-reperfusion are presented in Table 5B. The effects on the kidney tissue are presented in Table 5C.

### 3.4. Synthesis of the Results

#### 3.4.1. Qualitative Synthesis

**Figure 2:**
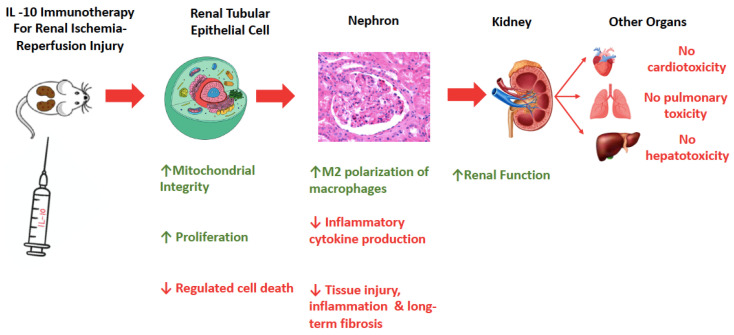
The effect of interleukin-10 immunotherapy on renal ischemia-reperfusion injury: qualitative synthesis of the systematic review.

Overall, seven in vivo studies corresponding to a population of 268 mice/rats were included in the present systematic review. The qualitative synthesis of the results of the included studies is summarized in Figure 2. The present systematic review has elucidated three levels of action of IL-10 immunotherapy in animal models of kidney ischemia-reperfusion injury. IL-10 immunotherapy acts on the tubular epithelial cell and preserves mitochondrial integrity [19,20], reduces regulated cell death (apoptosis and pyroptosis) [19,20,23] and promotes regeneration/proliferation [21,23]. In the kidney tissue, IL-10 immunotherapy promotes M2 polarization of the macrophages (anti-inflammatory and pro-repair phenotype) [20,23,25], decreases the production of inflammatory cytokines (TNF-α, IL-1β and IL-6), reduces the tissue injury [19,20,23] and the long-term fibrosis [20,21,22] following ischemia-reperfusion. At an organ level, IL-10 immunotherapy improves kidney function [19,20,22,23,24,25]. IL-10 immunotherapy has not been associated with cardiotoxicity, pulmonary toxicity or hepatotoxicity in animal models of renal ischemia-reperfusion injury [19,20,24].

#### 3.4.2. Quantitative Synthesis

Seven in vivo preclinical studies corresponding to a population of 268 mice/rats were included in the present meta-analysis.

##### Renal Function

**Figure 3:**
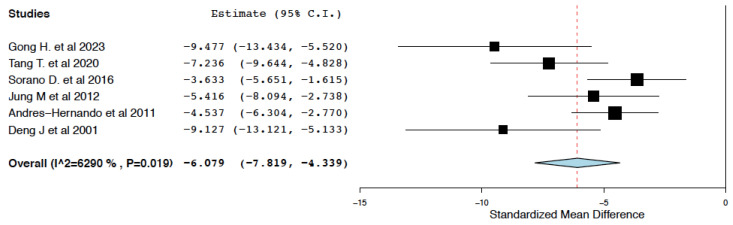
Serum creatinine: random effects meta-analysis (IL-10 immunotherapy vs. control treatment) [19,20,22,23,24,25].

**Figure 4:**
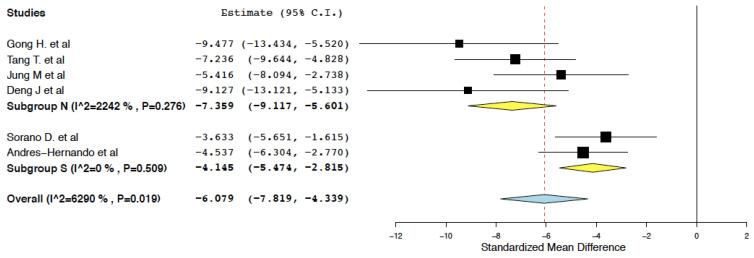
Serum creatinine: random effects subgroup meta-analyses in groups classified by WIT (IL-10 immunotherapy vs. control treatment) [19,20,22,23,24,25].

Compared to the control treatment, IL-10 immunotherapy was found to effectively reduce serum creatinine within 24 h of administration (95% CI: −7.819, −4.339, I^2^ = 62.9%) (Figure 3). This meta-analysis presented high heterogeneity (I^2^ = 62.9%). To explore this heterogeneity, subgroup meta-analyses were performed in groups classified by WIT. WIT ≥ 35 min was considered adequate. WIT < 35 min was considered as short and a risk factor for inadequate disease induction. In studies with WIT ≥ 35 min, IL-10 immunotherapy was again found to reduce serum creatinine in the immediate period following an ischemia-reperfusion insult (95% CI: −9.117, −5.061, I^2^ = 22.42%) (Figure 4).

##### Long-Term Fibrosis

**Figure 5:**
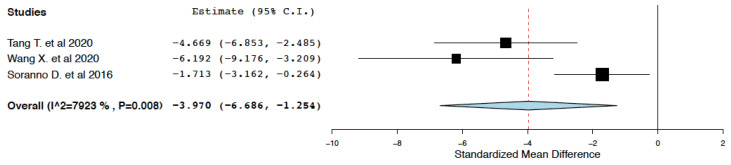
Kidney fibrosis: random effects meta-analysis (IL-10 immunotherapy vs. control treatment) [20,21,22].

**Figure 6:**
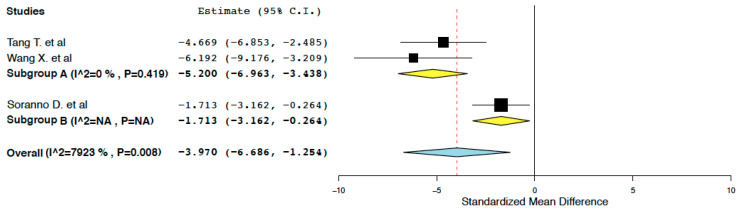
Kidney fibrosis: random effects subgroup meta-analyses in groups classified by form of IL-10 administered (IL-10 immunotherapy vs. control treatment) [20,21,22].

Compared to the control treatment, IL-10 immunotherapy was found to effectively reduce kidney fibrosis in the long-term period (7–28 days) following ischemia-reperfusion injury (95% CI: −6.686, −1.254 I^2^ = 79.23%) (Figure 5). This meta-analysis presented high heterogeneity (I^2^ = 79.23%). To explore this heterogeneity, subgroup meta-analyses were performed in groups classified by the form of IL-10 administered (IL-10 protein vs. IL-10 DNA/mRNA ± protein). Administration of IL-10 deoxyribonucleic acid (DNA) or IL-10 messenger ribonucleic acid (mRNA) was considered more likely to exert a sustained long-term effect in tissue fibrosis compared to the administration of the IL-10 protein alone. Compared to the control treatment, IL-10 DNA/mRNA administration was found to more effectively reduce kidney fibrosis in the long-term period following ischemia-reperfusion (95% CI: −6.963, −3.438, I^2^ = 0%) (Figure 6).

##### Tissue Integrity and Inflammation

**Figure 7:**
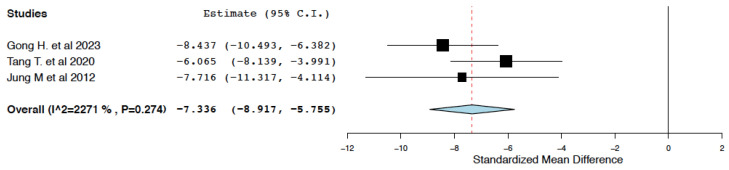
Tubular injury score: random effects meta-analysis (IL-10 immunotherapy vs. control treatment) [19,20,23].

Compared to the control treatment, IL-10 immunotherapy was found to reduce tubular injury score within 24 h of administration (95% CI: −8.917, −5.755, I^2^ = 22.71%) (Figure 7).

##### Regulated Cell Death

**Figure 8:**
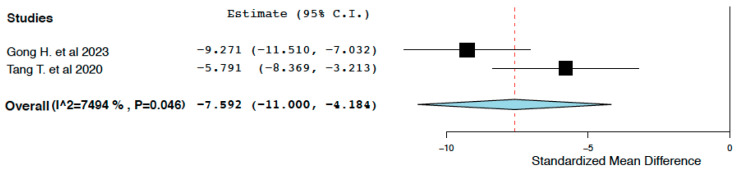
TUNEL positive cells/HPF: random effects meta-analysis (IL-10 immunotherapy vs. control treatment) [19,20].

Compared to the control treatment, IL-10 immunotherapy was found to reduce regulated cell death (TUNEL positive cells/HPF) within 24 h of administration (95% CI: −11.000, −4.184, I^2^ = 74.94%) (Figure 8). This meta-analysis presented high heterogeneity, but it was not possible to explore it further.

### 3.5. Risk of Bias across Studies

We performed a GRADE assessment to identify the risk of bias across studies. Since this is a meta-analysis of pre-clinical studies, our GRADE approach was modified, as per Wei D et al., to better capture the quality of evidence produced [10]. The GRADE assessment is presented in Table 6.

## 4. Discussion

### 4.1. Summary of Evidence

The present systematic review and meta-analysis of preclinical evidence included seven studies that correspond to a population of 268 mice and rats and credibly assessed the effect of IL-10 immunotherapy on renal ischemia-reperfusion injury. Our results suggest that IL-10 immunotherapy has the potential to safely improve outcomes of renal ischemia-reperfusion injury.

The primary endpoint for this study was kidney function following renal ischemia-reperfusion injury. IL-10 immunotherapy was found to effectively reduce kidney dysfunction in the immediate period following an ischemia-reperfusion insult (moderate quality of evidence).

There were multiple secondary endpoints to this study aiming to capture the adverse outcomes and the effects of IL-10 immunotherapy on the TEC and the renal tissue. No adverse outcomes (cardiotoxicity, pulmonary toxicity and hepatotoxicity) of IL-10 immunotherapy were captured (low quality of evidence). IL-10 immunotherapy was found to reduce regulated cell death in TECs (moderate quality of evidence). In the present systematic review, IL-10 immunotherapy was associated with preserved mitochondrial integrity and advanced regeneration in TECs (very low quality of evidence). IL-10 immunotherapy was found to reduce tissue injury and long-term fibrosis following renal ischemia-reperfusion (moderate quality of evidence). These effects were thought to be mediated by the M2 polarization of the local macrophages (anti-inflammatory and pro-repair phenotype) and by a decrease in the production of inflammatory cytokines (TNF-α, IL-1β and IL-6) (very low quality of evidence).

### 4.2. Comparison to Relevant Research

The evidence generated here is further supported by previous original research studies. In 2019, Kenji Sakai et al. investigated the role and the mechanisms of action of IL-10 in renal IRI by studying wild-type vs. IL-10 knockout mice. They found that in response to IRI, IL-10 knockout mice demonstrated worsening renal function (serum creatinine and BUN) and increased expression of markers of AKI (Kidney Injury Molecule-1—KIM-1), inflammatory chemokines (Regulated on Activation, Normal T cell Expressed and Secreted—RANTES), cytokines (IL-1β, IL-6, and interleukin-18—IL-18) and pro-apoptosis factors (Bax and caspase-3) [15]. These effects were reversed when recombinant IL-10 was administered in IL-10 knockout mice [15]. Xin Wan et al. used a similar methodology (IL-10-deficient mice) and had similar findings. In their conclusions, the authors stressed that in renal IRI, IL-10 acts through the suppression of inflammatory cytokines (TNF-α, IL-6) [12]. Daeman MA et al. subjected mice to renal ischemia and reperfusion with an anti-IL-10 antibody. They found that compared to the control group, mice treated with anti-IL-10 demonstrated worsening renal function and increased apoptosis in TECs [11]. Seung Hee Yang et al., in their 2011 study, found that sulfatide-reactive natural killer T cells ameliorate renal ischemia-reperfusion injury by reducing Acute Tubular Necrosis (ATN), partly through increased expression of IL-10 and attenuation of TECs’ apoptosis [26]. In 2018, Anja Thorenz et al. studied C5aR2 knockout out mice vs. wild-type mice in a model of renal ischemia-reperfusion injury [16]. The investigators observed that compared to wild-type mice, C5aR2 knockout out mice up-regulated IL-10 expression. The C5aR2 knockout out mice also had enhanced TEC proliferation with reduced long-term renal fibrosis. In their conclusions, the authors attributed these effects to the increased expression of IL-10 [16]. Michael J Eppinger et al. investigated the effect of IL-10 immunotherapy on lung ischemia-reperfusion injury. In their animal model, IL-10 immunotherapy reduced tissue (lung) injury and production of TNF-α [27]. In 2017, Mira Jung et al. investigated the effects of IL-10 immunotherapy in an animal model of myocardial infarction (MI) [28]. They found that IL-10 immunotherapy reduced post-MI inflammation through M2 polarization of the macrophages and enhanced cellular proliferation/regeneration [28]. IL-10 immunotherapy was also associated with reduced fibrosis in animal models of chronic liver disease [29].

### 4.3. Strengths and Limitations

This is the first systematic review and meta-analysis to credibly assess the effect of IL-10 immunotherapy on renal ischemia-reperfusion injury. Our review included exclusively pre-clinical animal studies. We assessed the risk of bias in the individual studies. The risk of selection bias was deemed unclear in four of the included studies. We qualitatively and quantitively synthesized the results of the identified studies. It was not possible to perform meta-analyses for all the secondary outcomes. Our meta-analyses demonstrated high heterogeneity. This heterogeneity was explored and dealt with, where possible. We were not able to explore the heterogeneity in the meta-analysis for TEC-regulated cell death (secondary outcome). To assess the risk of bias across the studies and the quality of the evidence generated, we followed the GRADE methodology. The evidence generated was deemed from very low certainty to moderate certainty. Clinical studies are needed to investigate the translational potential of IL-10 immunotherapy.

### 4.4. Future Research: Translational Potential

Our study sheds light on pathophysiological pathways involved in the immune regulation of renal ischemia-reperfusion injury. Our findings suggest translational potential for IL-10 immunotherapy. Importantly, our results hold significant implications for the fields of kidney disease prevention, kidney donation, and renal transplantation [30,31]. Here we demonstrated that IL-10 immunotherapies show potential to prevent the progression from renal ischemia-reperfusion injury to chronic kidney disease (CKD). Clinical studies are needed to explore the role of IL-10 immunotherapy in secondary kidney disease prevention. Our findings also suggest the capacity of IL-10 immunotherapy to optimize kidney viability. Future research is warranted to explore IL-10 immunotherapy’s role in reconditioning kidneys from extended criteria donors, thereby expanding the organ pool. Existing immunosuppression protocols contribute significantly to morbidity and mortality, particularly in the elderly population, and hinder kidney transplant function. Future studies are needed to evaluate IL-10 immunotherapies as potential post-transplant maintenance immunomodulatory treatments. Finally, in order to conduct clinical studies, future researchers need to overcome certain barriers. These include the instability of the recombinant IL-10 protein and the risks associated with the different delivery vehicles (extracellular vesicles, macrophages, etc.) [32]. Alternate methods of endogenously up-regulating IL-10 expression (i.e., small activating RNA (saRNA) up-regulation of IL-10) should, therefore, be considered.

## 5. Conclusions

This systematic review and meta-analysis of preclinical studies assessed the effect of IL-10 immunotherapy on renal ischemia-reperfusion injury. Our results suggest that IL-10 immunotherapy safely improves outcomes in animal models of renal ischemia-reperfusion. It reduces TEC-regulated cell death, kidney tissue injury, inflammation and fibrosis and improves renal function following renal IRI. The translational potential of IL-10 immunotherapy needs to be further investigated in clinical trials.

## Figures and Tables

**Table 1:** Systematic reviews’ inclusion and exclusion criteria.

Inclusion Criteria	Exclusion Criteria
Pre-clinical (in vivo and ex vivo) interventional studies. Clinical, prospective and retrospective interventional studies.	In vitro, observational or non-comparative studies; case reports, reviews, meta-analyses, commentaries and editorials.
Studies in animals or humans undergoing renal ischemia-reperfusion; studies in animal or human kidneys undergoing ischemia-reperfusion.	Studies that did not involve animal or human participants or animal/human kidneys undergoing ischemia-reperfusion.
Studies where any form of IL-10 was exogenously administered (IL-10 protein, IL-10 mRNA, IL-10 DNA) with any delivery vehicle/carrier.	Studies where IL-10 was not administered exogenously. Studies where the effect of IL-10 was studied through decreasing or knocking out the cytokine’s expression.
Studies where the main comparators were animal/human subjects or kidneys that did not receive exogenous administration of IL-10.	Studies with no controls.
Studies that reported renal function (serum creatinine) following treatment for renal IRI. Studies that reported mitochondrial integrity of TECs; TECs’ proliferation (including expression of ki-67 and PCNA); regulated TEC death (including TUNEL assay and expression/activity of caspase 3); expression of inflammatory cytokines (TNF-α, IL-1β and IL-6); M1/M2 macrophages polarization (including expression of iNOS and CD206); tissue integrity and inflammation (tubular injury score); long-term kidney fibrosis (fibrotic area %); and adverse outcomes (pulmonary toxicity, cardiotoxicity hepatotoxicity) following treatment for renal IRI.	Studies that did not report these outcomes.

**Table 2:** Risk of bias assessment.

Authors, Year	Selection Bias			Performance Bias		Detection Bias		Attrition Bias	Reporting Bias	Other Sources of Bias
	Sequence Generation	Baseline Characteristics	Allocation Concealment	Random Housing	Blinding	Random Outcome Assessment	Blinding	Missing Outcome Data	Selective Outcome Reporting	
Gong H. et al., 2023 [19]	Unclear Risk of Bias	Low Risk of Bias	Unclear Risk of Bias	Low Risk of Bias	Low Risk of Bias	Unclear Risk of Bias	Low Risk of Bias	Low Risk of Bias	Low Risk of Bias	None identified
Tang T. et al., 2020 [20]	Unclear Risk of Bias	Low Risk of Bias	Unclear Risk of Bias	Low Risk of Bias	Unclear Risk of Bias	Low Risk of Bias	Low Risk of Bias	Low Risk of Bias	Low Risk of Bias	None identified
Wang X. et al., 2020 [21]	Unclear Risk of Bias	Unclear Risk of Bias	Unclear Risk of Bias	Low Risk of Bias	Unclear Risk of Bias	Unclear Risk of Bias	Unclear Risk of Bias	Low Risk of Bias	Low Risk of Bias	None identified
Soranno D. et al., 2016 [22]	Unclear Risk of Bias	Unclear Risk of Bias	Unclear Risk of Bias	Low Risk of Bias	Unclear Risk of Bias	Low Risk of Bias	Low Risk of Bias	Low Risk of Bias	Low Risk of Bias	None identified
Jung M et al., 2012 [23]	Unclear Risk of Bias	Unclear Risk of Bias	Unclear Risk of Bias	Low Risk of Bias	Unclear Risk of Bias	Unclear Risk of Bias	Unclear Risk of Bias	Low Risk of Bias	Unclear Risk of Bias	None identified
Andres-Hernando et al., 2011 [24]	Unclear Risk of Bias	Low Risk of Bias	Unclear Risk of Bias	Low Risk of Bias	Unclear Risk of Bias	Low Risk of Bias	Low Risk of Bias	Low Risk of Bias	Low Risk of Bias	None identified
Deng J et al., 2001 [25]	Unclear Risk of Bias	Unclear Risk of Bias	Unclear Risk of Bias	Low Risk of Bias	Unclear Risk of Bias	Unclear Risk of Bias	Unclear Risk of Bias	Low Risk of Bias	Low Risk of Bias	None identified

**Table 3:** Study and population characteristics.

Authors, Year	Study Design	Study Subjects	Study Size	Kidney Ischemia-Reperfusion Induction Protocol	Warm Ischemia Time (min)	Study Groups
Gong H. et al., 2023 [19]	Pre-clinical, In vivo	*C57BL/6* mice (male, 8 weeks old, weight 20–22 g)	72	Midline laparotomy and bilateral renal pedicle clamping	45	1. Sham-operated mice
						**2. IRI + normal saline**
						3. IRI + IL-10
						**4. IRI + platelet shipped IL-10**
Tang T. et al., 2020 [20]	Pre-clinical, In vivo	*C57BL/6* mice (male, 8–10 weeks old, weight 20–22 g)	40	A. Midline laparotomy and bilateral renal pedicle clamping	35	A1. Sham-operated mice
						**A2. IRI + vehicle treatment**
						A3. IRI + IL-10 (low dose)
						**A4. IRI + IL-10 (high dose)**
			24	B. Midline laparotomy and left renal pedicle clamping	35	B1. Sham-operated mice
						**B2. IRI + vehicle treatment**
						B3. IRI + IL-10 (low dose)
						**B4. IRI + IL-10 (high dose)**
Wang X. et al., 2020 [21]	Pre-clinical, In vivo	*C57BL/6* mice (male, 8–10 weeks old)	18	Midline laparotomy and left renal pedicle clamping	40	1. Healthy controls
						**2. IRI only**
						**3. IRI + IL-10**
Soranno D. et al., 2016 [22]	Pre-clinical, In vivo	*C57BL/6* mice (8–10 weeks old)	36	Midline laparotomy and bilateral renal pedicle clamping	22	1. Healthy controls
						**2. IRI + normal saline**
						3. IRI + hyaluronic acid hydrogel under the left kidney capsule
						**4. IRI + IL-10 under the left kidney capsule**
						5. IRI + hyaluronic acid hydrogel + IL-10 under the left kidney capsule
						6. IRI + hyaluronic acid hydrogel + IL-10 subcutaneously
Jung M et al., 2012 [23]	Pre-clinical, In vivo	*Sprague-Dawley* rats (male)	45	Midline laparotomy and bilateral renal pedicle clamping	45	1. Sham-operated mice
						2. IRI only
						3. **IRI + BMDMs (vehicle treatment)**
						4. IRI + β-gal expressing macrophages
						**5. IRI + IL-10 expressing macrophages**
						6. IRI + IL-10 macrophages + Anti-Lcn2
						7. IRI + IL-10 macrophages + Anti-IgG
						8. IRI + IL-10 macrophages + DFO
						9. IRI + Anti-Lcn2
Andres-Hernando et al., 2011 [24]	Pre-clinical, In vivo	*C57BL/6* mice (8–10 weeks old, weight 20–25 g)	19	Midline laparotomy and bilateral renal pedicle clamping	22	**1. IRI + vehicle treatment**
						**2. IRI + IL-10**
Deng J et al., 2001 [25]	Pre-clinical, In vivo	*BALB/c* mice	14	Midline laparotomy and bilateral renal pedicle clamping	40	1. Sham-operated mice
						**2. IRI + vehicle treatment**
						**3. IRI + IL-10**

**Table 4:** Intervention characteristics.

Authors, Year	Target IL-10 Immunotherapy Intervention						Target Control Intervention
	Form of IL-10 Administered	Delivery Vehicles	Timing in Reference to Reperfusion (hours)	Route	Dose (μg)	Frequency	
Gong H. et al., 2023 [19]	Protein	Platelets	0	IV	0.216	Once only	IV normal saline (0.1 mL) once only
Tang T. et al., 2020 [20]	Protein + mRNA + DNA	RAW 264.7 cells (macrophages)	0	IV	2.2	Once a day for 3 days	IV vehicle treatment once a day for 3 days
Wang X. et al., 2020 [21]	DNA	Lentiviral vector	−72	Sabcapsular (left kidney)	_	Once only	Nil
Soranno D. et al., 2016 [22]	Protein	15 μL solution of 0.1% mouse serum albumin	72	Sabcapsular (left kidney)	5	Once only	Subcut normal saline (0.5 mL) once a day for five days
Jung M et al., 2012 [23]	DNA	BMDMs	1	IV	_	Once only	IV untreated BMDMs once only
Andres-Hernando et al., 2011 [24]	Protein	Not reported	0	IV	Not reported	Once only	IV vehicle treatment once only
Deng J et al., 2001 [25]	Protein	Not reported	0	IP	0.5	Once only	IV vehicle treatment (0.5 mL) once only

**Table 5:** (**A**) Renal function and adverse outcomes following IL-10 immunotherapy for renal ischemia-reperfusion injury. (**B**) Effect of IL-10 immunotherapy on the tubular epithelial cell following renal ischemia-reperfusion. (**C**) Effect of IL-10 immunotherapy on the kidney tissue following ischemia-reperfusion.

(A)										
Authors, Year	Renal Function in the Immediate Period Following Ischemia-Reperfusion				Long-Term Cardiotoxicity Following IL-10 Immunotherapy		Long-Term Pulmonary Toxicity Following IL-10 Immunotherapy		Long-Term Hepatotoxicity Following IL-10 Immunotherapy	
	**Serum Creatinine (Mean ± SD) (mg/dL)**									
	**IL-10 Immunotherapy**		**Control Treatment**							
Gong H. et al., 2023 [19]	1.07 ± 0.06		1.98 ± 0.11		Nil abnormal on histology (H&E staining)		Nil abnormal on histology (H&E staining)		Nil abnormal on histology (H&E staining). No change in LFTs	
Tang T. et al., 2020 [20]	0.28 ± 0.12		1.81 ± 0.26		Nil abnormal on histology (PAS staining) Immunohistochemistry: No difference in count of CD3 T cells and macrophages/HPF between experimental and control groups		Nil abnormal on histology (PAS staining) Immunohistochemistry: No difference in count of CD3 T cells and macrophages/HPF between experimental and control groups		Nil abnormal on histology (PAS staining). No difference in count of CD3 T cells and macrophages/HPF between experimental and control groups. No change in LFTs	
Wang X. et al., 2020 [21]	_		_		_		_		_	
Soranno D. et al., 2016 [22]	0.8 ± 0.05		1.25 ± 0.15		_		_		_	
Jung M et al., 2012 [23]	0.6 ± 0.1		1.2 ± 0.1		_		_		_	
Andres-Hernando et al., 2011 [24]	0.57 ± 0.08		0.88 ± 0.06		_		Compared to control treatment, IL-10 immunotherapy led to reduction in lung MPO activity and lung CXCL1		Compared to control treatment, IL-10 immunotherapy led to reduction in liver IL-6 mRNA	
Deng J et al., 2001 [25]	0.5 ± 0.1		1.8 ± 0.15		_		_		_	
(**B**)										
**Authors, Year**	**TECs’ Regulated Cell Death**						**TECs’ Proliferation**		**Mitochondrial Integrity**	
	**TUNEL-positive cells/HPF (mean ± SD)**				**Caspase-3**					
	**IL-10 Immunotherapy**		**Control Treatment**							
Gong H. et al., 2023 [19]	3 ± 0.3		10 ± 1		Compared to control treatment, IL-10 immunotherapy resulted in 2-fold reduction in caspace 3 expression in immunohistochemistry with anti-caspase-3 antibodies		_		Compared to control treatment, IL-10 immunotherapy resulted in less mitochondrial swelling, brightened matrix, and fragmented cristae (TEM).	
Tang T. et al., 2020 [20]	12 ± 2		28 ± 3		Compared to control treatment, IL-10 immunotherapy resulted in 2-fold reduction in caspace 3 expression (Western blotting-normalized to β-actin)		_		Compared to control treatment, IL-10 immunotherapy resulted in less mitochondrial swelling, brightened matrix, and fragmented cristae (TEM).	
Wang X. et al., 2020 [21]	_		_		_		Compared to the control group, IL-10 immunotherapy resulted in increased TECs in the cortex and the medulla		_	
Soranno D. et al., 2016 [22]	_		_		_		_		_	
Jung M et al., 2012 [23]	_		_		Compared to control treatment, IL-10 immunotherapy resulted in significant reduction in caspase 3 activity		Clear increase in TECs’ proliferation (ki-67 and PCNA stainning)		_	
Andres-Hernando et al., 2011 [24]	_		_		_		_		_	
Deng J et al., 2001 [25]	_		_		_		_		_	
(**C**)										
**Authors, Year**	**Expression of Inflammatory Cytokines Following IL-10 Immunotherapy**				**M1/M2 Macrophage Polarization Following IL-10 Treatment**		**Tubular Injury Score (Mean ± SD)**		**Long-Term Kidney Fibrosis (Fibrotic Area %: Mean ± SD)**	
	**TNF-α**	**IL-1β**		**IL-6**	**Expression of iNOS**	**Expression of CD206**	**IL-10 Immunotherapy**	**Control Treatment**	**IL-10 Immunotherapy**	**Control Treatment**
Gong H. et al., 2023 [19]	Decreased expression compared to control treatment	Decreased expression compared to control treatment		Decreased expression compared to control treatment	_	_	1.3 ± 0.2	3.5 ± 0.3	_	_
Tang T. et al., 2020 [20]	Decreased expression compared to control treatment	Decreased expression compared to control treatment		Decreased expression compared to control treatment	Decreased expression compared to control treatment	Increased expression compared to control treatment	1.3 ± 0.3	3.2 ± 0.3	10% ± 5%	50% ± 10%
Wang X. et al., 2020 [21]	_	_		_	_	_	_	_	20 ± 4%	40% ± 1%
Soranno D. et al., 2016 [22]	_	_		_	_	_	_	_	4% ± 2%	7% ± 1%
Jung M et al., 2012 [23]	Decreased expression compared to control treatment	Decreased expression compared to control treatment		_	Decreased expression compared to control treatment	_	2.1 ± 0.2	6.5 ± 0.7	_	_
Andres-Hernando et al., 2011 [24]	_	_		Decreased expression compared to control treatment	_	_	_	_	_	_
Deng J et al., 2001 [25]	Decreased expression compared to control treatment	_		_	Decreased expression compared to control treatment	_	_	_	_	_

**Table 6:** GRADE assessment of quality of evidence.

Evidence	Risk of Bias	Indirectness	Inconsistency	Imprecision	Publication Bias	GRADE Assessment
Il-10 immunotherapy improves renal function following renal IRI.	No (low)	Yes (moderate)	No (low)	Yes (moderate)	No (low)	Moderate
IL-10 immunotherapy for renal IRI is not associated with cardiotoxicity, pulmonary toxicity and hepatotoxicity.	No (low)	Yes (moderate)	Yes (moderate)	Yes (moderate)	No (low)	Low
Il-10 immunotherapy reduces renal tissue injury and inflammation following renal IRI.	No (low)	Yes (moderate)	No (low)	Yes (moderate)	No (low)	Moderate
Il-10 immunotherapy reduces long-term kidney fibrosis following renal IRI.	No (low)	Yes (moderate)	Yes (moderate)	No (low)	No (low)	Moderate
Il-10 immunotherapy reduces inflammatory cytokines production following renal IRI.	No (low)	Yes (moderate)	Yes (significant)	Yes (significant)	No (low)	Very Low
Il-10 immunotherapy increases M2 polarization of the local macrophages following renal IRI	No (low)	Yes (moderate)	Yes (significant)	Yes (significant)	No (low)	Very Low
Il-10 immunotherapy improves TECs’ mitochondrial integrity following renal IRI.	No (low)	Yes (moderate)	Yes (significant)	Yes (significant)	No (low)	Very Low
Il-10 immunotherapy increases TEC regeneration/proliferation following renal IRI.	No (low)	Yes (moderate)	Yes (significant)	Yes (significant)	No (low)	Very Low
Il-10 immunotherapy reduces TEC-regulated death following renal IRI.	No (low)	Yes (moderate)	Yes (moderate)	No (low)	No (low)	Moderate

## Data Availability

No new data were generated. The data presented in the study are openly available in the source materials.

## Supplementary Materials

The supporting information can be downloaded at: https://www.mdpi.com/article/10.3390/ijms25116231/s1.

## Abbreviations

AKI	Acute Kidney Injury
ATN	Acute Tubular Necrosis
β-gal	β-galactosidase
BMDMs	Bone Marrow-Derived Macrophages
BUN	Blood Urea Nitrogen
CAMARADES	Collaborative Approach to Meta-Analysis and Review of Animal Data from Experimental Studies
CD206	Cluster of Differentiation 206
CI	Confidence Interval
CKD	Chronic Kidney Disease
DFO	DesFerrOxamine
DNA	DeoxyriboNucleic Acid
GRADE	Grading of Recommendations Assessment, Development and Evaluation
H&E	Hematoxylin and Eosin
HPF	High Power Field
IDMS	Isotope Dilution liquid chromatography–Mass Spectrometry
IFTA	Interstitial Fibrosis and Tubular Atrophy
IgG	Immunoglobulin G
IL-1β	Interleukin-1β
IL-10	Interleukin-10
IL-6	Interleukin-6
iNOS	inducible Nitric Oxide Synthase
IP	IntraPeritoneal
IRI	Ischemia-Reperfusion Injury
IV	IntraVenous
KIM-1	Kidney Injury Molecule-1
Lcn2	lipocalin-2
MI	Myocardial Infraction
MPO	MyeloPreOxidase
mRNA	messenger RiboNucleic Acid
PAS	Periodic Acid–Schiff
PCNA	Proliferating Cell Nuclear Antigen
PICO	Population, Interventions, Comparison, Outcomes
PRISMA	Preferred Reporting Items for Systematic Reviews and Meta-Analyses
RANTES	Regulated on Activation, Normal T cell Expressed and Secreted
RoB	Risk of Bias
saRNA	small activating RiboNucleic Acid
SD	Standard Deviation
SYRCLE	Systematic Review Centre for Laboratory Animal Experimentation
TECs	Tubular Epithelial Cells
TEM	Transition Electron Microscopy
Th1	T helper type 1
Th2	T helper type 2
TNF-α	Tissue Necrosis Factor α
TUNEL	Terminal deoxynucleotidyl transferase dUtp Nick-End Labelling
WIT	Warm Ischemia Time
