# Supplementary material for: The Effect of Interleukin-10 Immunotherapy on Renal Ischemia-Reperfusion Injury: A Systematic Review and Meta-Analysis of Preclinical Studies
Source: Int J Mol Sci. 2024 Jun 5;25(11):6231. doi: 10.3390/ijms25116231 (PMC11172821; doi:10.3390/ijms25116231)
Supplement: Supplementary file 1 [file ijms-25-06231-s001.zip › ijms-2992583-supplementary.pdf]

Table S1. Embase Search up to 31st of March 2023.	
1.	kidney allograft/ or kidney failure/ or kidney preservation/ or chronic kidney failure/ or kidney tissue/ or kidney/ or kidney fibrosis/ or acute kidney failure/ or kidney ischemia/ or kidney dysfunction/ or kidney transplantation/
2.	ischemia reperfusion injury.mp. or reperfusion injury/
3.	ischemia time/ or warm ischemia time/ or ischemia.mp. or kidney ischemia/ or cold ischemia time/ or ischemia/ or cold ischemia/
4.	reperfusion injury/ or renal ischemia reperfusion injury/ or reperfusion.mp. or reperfusion/
5.	Interleukin 10.mp. or interleukin 10/
6.	IL-10.mp. or interleukin 10/
7.	IL10.mp.
8.	IL-10 protein.mp.
9.	IL10 protein.mp.
10.	IL-10 mRNA.mp.
11.	IL10 mRNA.mp.
12.	IL-10 gene.mp.
13.	IL10 gene.mp.
14.	recombinant interleukin 10/ or IL-10 administration.mp.
15.	IL10 administration.mp.
16.	IL-10 up regulation.mp.
17.	IL-10 upregulation.mp.
18.	IL10 up regulation.mp.
19.	IL10 upregulation.mp.
20.	2 or 3 or 4
21.	5 or 6 or 7 or 8 or 9 or 10 or 11 or 12 or 13 or 14 or 15 or 16 or 17 or 18 or 19
22.	1 and 20 and 21
